# *Mycobacterium fortuitum* and Polymicrobial Peritoneal Dialysis-Related Peritonitis: A Case Report and Review of the Literature

**DOI:** 10.1155/2014/323757

**Published:** 2014-06-16

**Authors:** Anwar Hamade, Agnieszka Pozdzik, O. Denis, Monika Tooulou, Caroline Keyzer, F. Jacobs, Jose Khabbout, Joëlle L. Nortier

**Affiliations:** ^1^Department of Nephrology, Dialysis and Renal Transplantation, Erasme Hospital, Brussels 1070, Belgium; ^2^Department of Nephrology, Lebanese University, Beirut, Lebanon; ^3^Laboratory of Experimental Nephrology, Department of Biochemistry, Faculty of Medicine, Université Libre de Bruxelles, Brussels 1070, Belgium; ^4^Department of Microbiology, Erasme Hospital, Université Libre de Bruxelles, Brussels 1070, Belgium; ^5^Department of Radiology, Erasme Hospital, Université Libre de Bruxelles, Brussels 1070, Belgium; ^6^Department of Infectious Diseases, Erasme Hospital, Université Libre de Bruxelles, Brussels 1070, Belgium

## Abstract

*Mycobacterium fortuitum* is a ubiquitous, rapidly growing nontuberculous mycobacterium (NTM). It is the most commonly reported NTM in peritoneal dialysis (PD) associated peritonitis. We report a case of a 52-year-old man on PD, who developed refractory polymicrobial peritonitis necessitating PD catheter removal and shift to hemodialysis. Thereafter, *M. fortuitum* was identified in the PD catheter culture and in successive cultures of initial peritoneal effluent and patient was treated with amikacin and ciprofloxacin for six months with a good and sustained clinical response. Months after completion of the course of antibiotics, the patient successfully returned to PD. To our knowledge, this is the first reported case of *M. fortuitum* peritonitis in the field of polymicrobial PD peritonitis. It demonstrates the diagnostic yield of pursuing further investigations in cases of refractory PD peritonitis. In a systematic review of the literature, only 20 reports of *M. fortuitum* PD peritonitis were identified. Similar to our case, a delay in microbiological diagnosis was frequently noted and the Tenckhoff catheter was commonly removed. However, the type and duration of antibiotic therapy varied widely making the optimal treatment unclear.

## 1. Introduction

Bacterial peritonitis is the most common complication of peritoneal dialysis (PD) and often the reason for discontinuing this modality of renal replacement therapy. The vast majority of PD associated peritonitis cases are caused by aerobic bacteria, such as coagulase-negative staphylococci,* Staphylococcus aureus, *and* Pseudomonas aeruginosa*. Culture-negative peritonitis accounts for up to 30% of peritonitis cases [1]. Potential pathogens for this “culture-negative” peritonitis include mycobacteria and fungi. Failure to consider mycobacterial infection in the differential diagnosis of peritonitis may lead to delayed diagnosis and treatment, even to failure of PD. We describe the first case of NTM PD peritonitis caused by* Mycobacterium fortuitum *(*M. fortuitum*) in the field of polymicrobial PD peritonitis and a brief literature review concerning* M. fortuitum *peritonitis in PD patients.

## 2. Case Presentation

A 52-year-old male patient with hypertension, diabetes, and end-stage renal disease due to diabetic nephropathy had been treated with automated peritoneal dialysis for 2 years without complications. He reported adherence to his aseptic technique and had no previous history of peritonitis.

**Figure 1:**
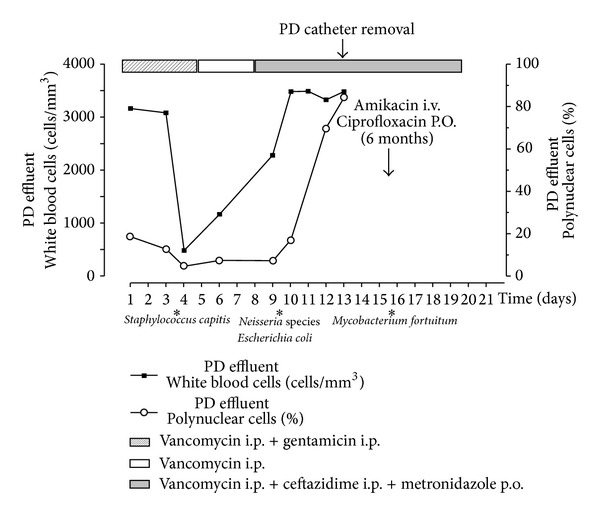
Time course of antibiotherapy and PD effluent parameters.

In March 2012, he presented with abdominal pain and turbid peritoneal fluid. Upon admission, the patient had a blood pressure of 120/80 mmHg and showed no signs of fever. Physical examination revealed soft abdomen with crust formation and erythema surrounding the exit site and no signs of catheter tunnel infection. Analyses of the peritoneal fluid demonstrated 740 white blood cells (WBC)/mm^3^ (predominantly polymorphonuclear neutrophils) (Figure 1). The peritoneal effluent cultures were negative and the exit site swab culture grew* Staphylococcus capitis*. The patient was empirically treated with intraperitoneal vancomycin and gentamicin. According to the bacterial sensitivity of* S. capitis, *gentamicin was stopped after 5 days and the patient was discharged from the hospital on intermittent intraperitoneal vancomycin treatment (Figure 1).

**Figure 2:**
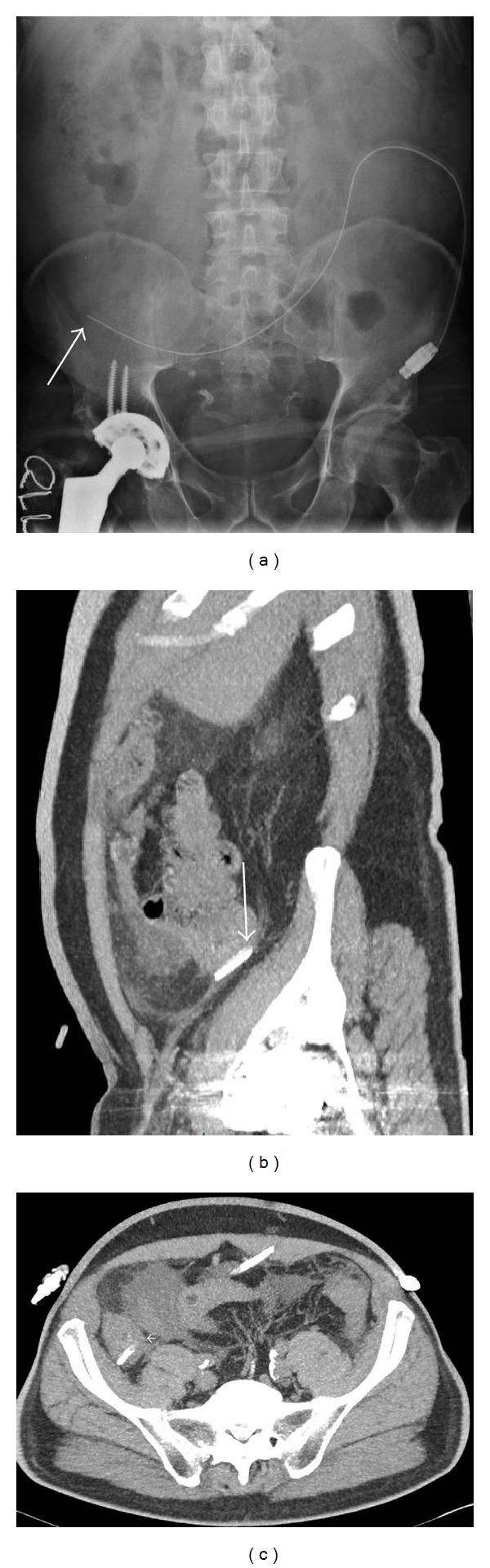
Radiologic findings. (a) Abdominal X-ray showing the PD catheter tip position in the right iliac fossa (white arrow). (b) Computerised tomography of the abdomen, showing the catheter tip resting in close contact with the pelvic wall and the colon (white arrow). (c) Computerised tomography of the abdomen, showing inflamed fat and bowel wall thickening (small white arrow).

Few days later, the patient was readmitted for persisting abdominal pain. He had fever; his arterial blood pressure was 130/80 mmHg with a heart rate of 85 beats/minute. Physical examination revealed mild abdominal distension with right iliac fossa tenderness to palpation; bowel sounds were present. Tenckhoff catheter exit site showed mild erythema. Abdominal CT scan demonstrated the PD catheter tip resting against the pelvic wall and the colon with inflamed fat and bowel wall thickening (Figure 2). The peritoneal fluid remained turbid (2200 WBC/mm^3^). The peripheral leukocyte count was 10.700/mm^3^, with a normal differential. The patient was put on ceftazidime and metronidazole in addition to vancomycin (i.p.). Dialysate culture grew* Escherichia coli, Neisseria *spp. Following treatment guidelines of refractory peritonitis and considering the risk of bowel perforation, the peritoneal catheter was surgically removed. One day later, the patient's temperature returned to normal and hemodialysis was started. The culture of the PD catheter and successive cultures of the initial peritoneal effluent grew* M. fortuitum*. Amikacin (1/48 hrs I.V.) and oral ciprofloxacin (250 mg, twice daily) were followed for six months with a good and sustained clinical response (Figure 1). Six months after completion of the course of antibiotics, a Tenckhoff catheter was reinserted and the patient successfully returned to PD. Seven months later, he received a cadaveric kidney transplant and he is doing very well.

## 3. Discussion

*Mycobacterium fortuitum* is the most commonly reported NTM associated with infection in PD patients. It belongs to group 4 (Runyon's classification) of rapidly growing NTM. These ubiquitous bacteria can be isolated from a number of natural sources including soil, dust, and water [2]. Relapsing and culture-negative peritonitis is typical. Guidelines of the International Society for Peritoneal Dialysis (ISPD) recommend that a negative dialysate culture at 3 days together with ongoing clinical evidence of peritonitis calls for specialized cultures for atypical causes of peritonitis. In case of a clinical suspicion of infection with* Mycobacterium* spp., repeated smears and centrifuge of effluent sediment with a combination of solid- and fluid-medium culture are suggested [3]. NTM PD peritonitis episodes respond neither to antibiotics typically prescribed for bacterial peritonitis nor to antituberculous medications. It is important that clinicians maintain a high level of suspicion for NTM peritonitis when PD associated peritonitis cases are culture negative or refractory to standard antibiotic treatment. The failure to consider mycobacterial infection in the differential diagnosis of peritonitis may lead to delayed diagnosis and treatment.

We conducted a systematic review of the literature focusing on* M. fortuitum *PD-related peritonitis. Twenty reports were identified. Five patients were women and 15 were men with an age ranging between 15 and 83 years. Duration of PD prior to* M. fortuitum *peritonitis ranged from a few days to 8 years. Fever and abdominal pain were the predominant clinical features at presentation. A delayed microbiological diagnosis was seen in almost all reports.* M. fortuitum *infection showed a propensity for abscess formation and tunnel infection (as reported in 7 of the 20 cases described). The Tenckhoff catheter was removed in all but 2 of the reported cases. The duration of the antibiotic therapy varied widely from 1 week to 12 months. The more common antibiotics used included amikacin (*n* = 10), clarithromycin (*n* = 6), ciprofloxacin (*n* = 5), and doxycycline (*n* = 4). Combined agents were commonly prescribed. Three deaths were reported in patients with* M. fortuitum *PD peritonitis. The main characteristics of the clinical cases reported in the literature are summarized in Table 1 [2, 4–16].

The safety and timing for attempted reinsertion of Tenckhoff catheters after treatment, and technique survival, are incompletely reported. The ISPD guidelines recommend consideration of catheter removal following a diagnosis of mycobacterial peritonitis although data supporting this recommendation are limited [3].

In our case, we noticed that the peritoneal dialysis catheter was removed because of the refractory PD peritonitis well before the identification of* M. fortuitum*. The contact of the intra-abdominal extremity of PD catheterwith the colon could be involved in an enteric bacterial leak to the peritoneum. The presence of* M. fortuitum* in the PD effluent culture is most likely secondary to PD catheter colonization. The environmental peritoneal catheter infection by* M. fortuitum *via the exit site has been reported [17]. In our case, this hypothesis was not confirmed as identification of* M. fortuitum* on the exit site culture was not done.

NTM are a rare but serious cause of PD peritonitis with high rates of Tenckhoff removal and conversion to hemodialysis. The literature review has shown that* M. fortuitum* was usually sensitive to amikacin, quinolones, and imipenem [18]. We advocate that optimal treatment of this infection is PD catheter removal and antibiotic therapy for at least 6 months after eradication of the bacteria.

## Figures and Tables

**Table 1:** *M. fortuitum* PD-related peritonitis: patient characteristics, antibiotic therapies, and outcome.

Case [ref.]	Age (years)/sex	Cause of ESKD	Time on PD (months)	Antibiotic therapy	Treatment duration	PD catheter removal	Outcome
1 [4]	32/M	NA	NA	Amikacin, tetracycline ciprofloxacin	Months	Yes	Resistant infection

2 [5]	42/M	DN	NA	Amikacin, doxycycline	20 days	Yes	Recovery

3 [5]	40/M	DN	24	Amikacin doxycycline	21 days	Yes	Death

4 [6]	16/M	SLE	36	Ciprofloxacin Trimethoprim Sulfamethoxazole	Months	Yes	Recovery

5 [7]	35/M	GN	10	Amikacin	NA	Yes	Recovery

6 [8]	71/M	GN	10	Amikacin Clarithromycin Trimethoprim Sulfamethoxazole	3 months	Yes	Recovery

7 [2]	83/F	NS	11	Ciprofloxacin Clofazimine	3 months	No	Recovery

8 [2]	61/M	GN	0.33	Amikacin	1 week	Yes	Recovery

9 [2]	50/M	GN	96	Imipenem Amikacin Sulfamethoxazole	NA	Yes	Improved

10 [9]	33/M	HK	NA	Clarithromycin Bactrim	6 months	Yes	Improved

11 [9]	71/F	AED	<1	Amikacin Bactrim Clarithromycin	3 months	Yes	Death

12 [10]	45/F	NA	36	Isoniazid Rifampin Ethambutol	6 months	Yes	Recovery

13 [11]	65/M	NA	60	Levofloxacin Clarithromycin	12 months	No	Recovery

14 [12]	59/M	NS	1	Vancomycin Doxycycline	1 month	Yes	Recovery

15 [12]	65/M	DN	NA	Minocycline	6 days	Yes	Recovery

16 [13]	38/F	HUS	6	Moxifloxacin, clarithromycin, and doxycycline	NA	Yes	Recovery

17 [14]	62/M	DN	9	Cefoxitin, clarithromycin	Weeks	Yes	Recovery

18 [15]	47/M	DN	6	Clarithromycin, meropenem, ciprofloxacin	2 months	Yes	Recovery

19 [8]	68/F	DN	48	Amikacin, ciprofloxacin	6 months	Yes	Death

20 [16]	15/M	NA	NA	Amikacin, cefoxitin	NA	Yes	NA

AED: atheroembolic disease; DN: diabetic nephropathy; ESKD: end-stage kidney disease; GN: glomerulonephritis; HK: horseshoe kidney; HUS: hemolytic uremic syndrome; NA: not available; NS: hypertensive nephrosclerosis; PD: peritoneal dialysis; SLE: lupus nephritis.

## Abbreviations

AED: Atheroembolic disease
DN: Diabetic nephropathy
ESKD: End-stage kidney disease
GN: Glomerulonephritis
HK: Horseshoe kidney
HUS: Hemolytic uremic syndrome
NA: Not available
NS: Hypertensive nephrosclerosis
PD: Peritoneal dialysis
SLE: Lupus nephritis.
